# Theory-Based Digital Interventions to Improve Asthma Self-Management Outcomes: Systematic Review

**DOI:** 10.2196/jmir.9666

**Published:** 2018-12-12

**Authors:** Helen J Lycett, Eva M Raebel, Emilie K Wildman, Jordi Guitart, Thomas Kenny, Jon-Paul Sherlock, Vanessa Cooper

**Affiliations:** 1 Spoonful of Sugar Ltd London United Kingdom; 2 Pharmaceutical Technology & Development AstraZeneca Macclesfield United Kingdom; 3 UCL School of Pharmacy University College London London United Kingdom

**Keywords:** asthma, adherence, self-management, quality of life, digital interventions, psychological theory

## Abstract

**Background:**

Asthma is a chronic disease requiring effective self-management to control it and prevent mortality. The use of theory-informed digital interventions promoting asthma self-management is increasing. However, there is limited knowledge concerning how and to what extent psychological theory has been applied to the development of digital interventions, or how using theory impacts outcomes.

**Objective:**

The study aimed to examine the use and application of theory in the development of digital interventions to enhance asthma self-management and to evaluate the effectiveness of theory-based interventions in improving adherence, self-management, and clinical outcomes.

**Methods:**

Electronic databases (CENTRAL, MEDLINE, EMBASE, and PsycINFO) were searched systematically using predetermined terms. Additional studies were identified by scanning references within relevant studies. Two researchers screened titles and abstracts against predefined inclusion criteria; a third resolved discrepancies. Full-text review was undertaken for relevant studies. Those meeting inclusion criteria were assessed for risk of bias using the Cochrane Collaboration tool. The review was conducted in accordance with the Preferred Reporting Items for Systematic Reviews and Meta-Analyses statement. Study outcomes were classified as medication adherence, self-management, asthma control, clinical markers of health, quality of life, other quality of life outcomes, and health care utilization. Effectiveness was calculated as an average outcome score based on the study’s reported significance. The Theory Coding Scheme (TCS) was used to establish the extent to which each intervention had applied theory and which theoretical constructs or behavioral determinants were addressed. Associations between TCS scores and asthma outcomes were described within a narrative synthesis.

**Results:**

Fourteen studies evaluating 14 different digital interventions were included in this review. The most commonly cited theories were Social Cognitive Theory, Health Belief Model, and Self-Efficacy Theory. A greater use of theory in the development of interventions was correlated with effective outcomes (*r*=.657; *P*=.01): only the 3 studies that met >60% of the different uses of theory assessed by the TCS were effective on all behavioral and clinical outcomes measured. None of the 11 studies that met ≤60% of the TCS criteria were fully effective; however, 3 interventions were partially effective (ie, the intervention had a significant impact on some, but not all, of the outcomes measured). Most studies lacked detail on the theoretical constructs and how they were applied to the development and application of the intervention.

**Conclusions:**

These findings suggest that greater use of theory in the development and application of digital self-management interventions for asthma may increase their effectiveness. The application of theory alone may not be enough to yield a successful intervention, and other factors (eg, the context in which the intervention is used) should be considered. A systematic approach to the use of theory to guide the design, selection, and application of intervention techniques is needed.

## Introduction

### Background

Approximately 235 million people worldwide are living with asthma [1]. First-line treatment for this chronic disease consists of a combination of quick-reliever inhalers (short-acting beta-agonists) during exacerbations and daily use of preventer medication (mainly inhaled corticosteroids, ICS) to control the disease [2]. Asthma is usually self-managed at home by the patient or caregivers [3], therefore, its effective control depends upon the patient’s behavior [4,5].

Efficient self-management involves active commitment to follow a written asthma action plan, self-monitoring symptoms, controlling environmental factors and, importantly, adhering to treatment [5-7]. Adherence to medication is a major determinant of treatment success in long-term conditions [8,9]. An adherence rate to ICS of >80% is needed to reduce asthma exacerbations [10], successfully control symptoms, and improve lung function [9,11]. This level of adherence has also been shown to decrease hospital admissions by 30% [9].

Despite these benefits, adherence rates to asthma treatment remain low [12] and variable [13]. In general, 30% to 70% of people on long-term preventer therapy do not maintain the high levels of adherence necessary for good asthma control. Suboptimal levels of adherence are found in adults [11], children [14,15], and adolescents [14-16].

Effective self-management of asthma is dependent on multiple factors, including consideration of patients’ perceptual and practical barriers to their disease and treatment [4]. Patients adopt self-management and adherence behaviors to cope with their illness, and these are influenced by their perceptions of their condition [17]. Nonadherence to asthma medication is influenced by perceptual barriers such as patients’ doubts about their need for treatment and treatment concerns (eg, fears about possible short- or long-term effects of treatment [18]) and/or as a result of practical barriers (eg, forgetting, bad inhaler technique).

Inadequate adherence to preventer medication can lead to overuse of relievers and the prescription of higher doses of medication than the patient needs [9]. Nonadherence has been associated with uncontrolled asthma, poor clinical outcomes, increased hospitalizations, decreased quality of life, absenteeism from work/school, and mortality in adults and children [8,19-21]. Most patients do not inform their health care professional when they stop treatment [8,22]; therefore, there may be limited opportunities to support patients to get the most from their medicines.

There is a clear need for effective self-management interventions, yet, to date, interventions have had varying degrees of success [23]. Digital support services (mobile and Web technologies) may increase the accessibility of interventions, given that most people now use electronic devices in their daily lives [24] and are willing to self-manage their disease using mobile technology interventions [25]. Digital support services can be highly scalable, personalized to increase medication adherence in targeted patient populations, can be applied in real time, and have the potential to provide consistency and delivery at low cost.

Digital adherence interventions, from electronic monitoring to short message service (SMS)–based programs, have been evaluated across long-term conditions with varying degrees of success [26-28]. However, the literature has been dominated by small-scale feasibility and exploratory studies and pilot evaluations that lack statistical power [26,29]. For patients with asthma, digital support services may provide a highly accessible and effective means of monitoring and improving adherence to treatment and disease control.

Recent systematic reviews have found that digital interventions can improve adherence to asthma preventer medication and asthma control when compared with standard treatment [12,30,31]. Miller et al [12] conducted a recent review and meta-analysis of mobile health (mHealth) interventions for the self-management of asthma comparing mHealth interventions with usual care and found a moderate effect on adherence, a large effect on quality of life, but no significant effect on lung function. The authors also found mHealth interventions to be as effective as paper-based monitoring on adherence and clinical outcomes. However, the findings of individual studies have been inconsistent. Although telemonitoring (text messaging, Web systems, etc) was not associated with better control of asthma symptoms when compared with usual care [32], internet-based self-management support has been shown to improve asthma quality of life and asthma control [33].

Guidelines for the development of interventions recommend the use of a theoretical framework or model of behavior change [34-37]. Theory can be used in various ways, for example, to identify modifiable determinants of health behaviors to be addressed within interventions (eg, illness perceptions), to select appropriate techniques to address behavioral determinants (eg, motivational interviewing), or to select people who are most likely to benefit from the intervention (eg, patients who have misconceptions about their illness or treatment). Many theory-based interventions used to explain health behavior have been based on social cognition theories [37,38]. These include Social Cognitive Theory (SCT) [39], the Health Belief Model (HBM) [40], Theory of Reasoned Action (TRA) [41], and Theory of Planned Behavior (TPB) [42], all of which are based on the premise that people are rational decision makers who can weigh up the advantages and disadvantages of adopting a behavior.

Several reviews of behavior change interventions have shown that interventions that explicitly refer to a theoretical approach to their development are more effective than those that lack a theoretical base [43-45]. A systematic review of interventions to improve adherence to asthma medicines showed that the use of theory was more common among effective than ineffective interventions [46], and another study reviewed the application of behavior change theory and clinical guidelines on internet-based asthma interventions [47]. However, these reviews only indicated whether theory was cited within the paper, rather than the extent to which theory was used to guide the development of the intervention or its effect on clinical outcomes. A review of digital interventions across long-term conditions found that more extensive use of theory was associated with a larger effect on health-related behavior [48]. To date, no systematic reviews of asthma self-management interventions have assessed how the use of theory impacts their effectiveness; therefore, little is known about how and to what extent theory has been applied, which theoretical models show promise, or which components of these models are most effective.

### Objectives

This review was designed to address the following questions about how best to use theory in the development of digital self-management interventions for asthma: (1) are theory-based digital interventions to enhance asthma self-management effective at changing behavior and improving clinical outcomes and quality of life?; (2) which theories have been applied to the development of digital interventions to enhance asthma self-management, and which theoretical constructs and behavioral determinants have been addressed?; (3) how and to what extent have theoretical models been applied to the development of digital interventions to enhance asthma self-management?

## Methods

### Literature Search

Searches were conducted using CENTRAL (The Cochrane Library), MEDLINE, EMBASE, and PsycINFO. Predetermined terms within titles, abstracts, and keywords were used to identify relevant studies. More detailed information about search terms used is available in Multimedia Appendix 1 Searches were completed on June 22, 2017. This systematic review was conducted in accordance with the Preferred Reporting Items for Systematic Reviews and Meta-Analyses (PRISMA) statement [49].

### Selection of Papers

**Figure 1:**
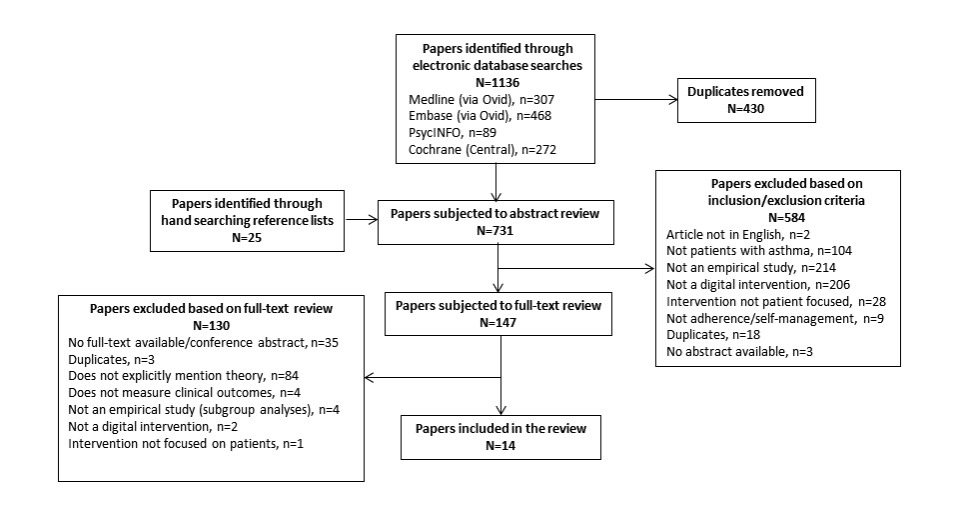
Preferred Reporting Items for Systematic Reviews and Meta-Analyses (PRISMA) flow diagram of the selection process of studies included in the review.

Titles, abstracts, and keywords from the electronic searches were screened independently by 2 researchers (HJL, EKW) and coded as “include” or “exclude” with both researchers screening all studies (100% overlap). Discrepancies were resolved by a third researcher (VC). Full texts of relevant papers were subjected to further scrutiny, and reference lists within relevant papers were hand-searched for significant titles, which were screened following the same process above. Final papers were selected based on the inclusion and exclusion criteria presented in Textbox 1. The selection process of papers for the review is summarized in Figure 1.

### Quality Assessment

The Cochrane Collaboration tool [50] was used to assess bias in the studies reporting on randomized controlled trials (RCTs). The item *blinding participants and personnel* was excluded because it would not be possible to blind participants to the use of the digital intervention. Each of the remaining 6 items was rated independently (low/high/unclear) by 2 researchers (HJL, EKW). Any disagreements were resolved through discussion.

### Data Extraction and Synthesis

#### Study Characteristics

Data were extracted by 2 independent researchers (HL, EKW). Data extracted on characteristics of the interventions included country, study design (RCT or pre-post design), inclusion criteria of participants, sample size, percentage of females, and mean age (or range). Details can be found in Multimedia Appendix 2.

#### Mode of Digital Delivery

Interventions were classified as fully digital or partly digital (digital and nondigital components). Data were extracted on the type of digital platform (eg, SMS, smart device app) and the type of nondigital component (eg, telephone call, paper-based). Full details are available in Multimedia Appendix 3.

#### Outcomes

To be able to compare the efficacy of the interventions on self-management, and as studies reported on different outcomes, only outcomes relevant to the study were extracted (EKW, EMR; eg, knowledge was not included) and classified under one of these overall themes: adherence to medication, self-management and asthma control, clinical markers of health, quality of life, other quality of life outcomes and health care utilization (Table 1). The intervention was considered to be effective on a specific outcome if the study reported a statistically significant (*P*<.05) improvement in the outcome. This included a significantly improved outcome in the intervention group relative to the control group for RCTs or a significant positive change in the outcome in pre-post studies. A score based on the study’s reported significance level was assigned to each outcome (2=if reported as a significant *P* value, 1=if reported as a marginally significant *P* value, and 0=if reported as not significant). An average score was applied when different suboutcomes of the same outcome were reported in the same study (eg, both symptom days and symptom nights were reported as clinical markers of health [51]). Finally, an average score was calculated for each study by adding the average outcome scores and dividing this result by the total number of outcomes. Therefore, interventions were deemed to be fully effective if they were associated with an outcome average score of 2.0, partially effective if they were in the range of 1.0 to 1.9, and not effective if the score was in the range of 0 to 0.9.

**Inclusion and exclusion criteria.**

**Inclusion criteria**

- Paper in English
- Patients with asthma
- Empirical study (pilot, feasibility, or evaluative study)
- Intervention focused on patient (rather than physician or carer)
- Digital intervention (eg, online intervention, smart phone app, electronic monitor, short message service (SMS), interactive voice recognition, or wearable
- Intervention designed to enhance adherence or persistence with asthma medication or self-management
- Explicit mention of the use of theory to design the self-management intervention or to increase engagement with the intervention

**Exclusion criteria**

- Conference abstracts
- Paper not in English
- Review or letter
- Intervention is delivered to parent(s) of children with asthma
- Not an empirical study
- Clinician focus (clinician attitude, behavior, or diagnostic tool)
- Intervention not designed to enhance self-management or adherence or persistence with asthma medication
- Intervention was not electronic
- Full-text paper not available

**Table 1:** Application of theory according to the Theory Coding Scheme (TCS) and effectiveness scores for study outcomes.

Authors, year	N	Behavior change model/theory	Theory Coding Scheme (Item number)	% theory applied	Adherence	Self-management and control	Clinical markers of health	Quality of life	Other Quality of life outcomes	Health care utilization	Outcomes average score
Bartholomew, 2000 [54]	133	SRM^a^; SCT^b^	1; 3; 5; 6	36	N/A^c^	2	2	N/A	N/A	1	1.67
Bartlett, 2002 [62]	16	SLT^d^	1; 2; 3; 5; 6; 7; 8; 9	73	2	N/A	N/A	N/A	N/A	N/A	2
Huss, 2003 [56]	101	PRECEDE-PROCEED model; Developmental; Social Support and learning theories	1; 2; 5	27	N/A	N/A	0	0	N/A	N/A	0
Krishna, 2003 [58]	228	SRT^e^	1	9	N/A	N/A	1.33	0	1	0.67	0.78
Joseph, 2007 [57]	315	Transtheoretical Model; HBM^f^	1; 2; 5; 6; 11	45	1	N/A	2	0	1.33	1.5	1.17
Bender, 2010 [55]	50	Benefit-Risk Model of Health Behavior	1; 2; 3; 5	36	2	0	N/A	0	N/A	N/A	0.68
Petrie, 2012 [60]	148	Extended SRM	1; 2; 3; 4; 5; 6; 7;11	73	2	N/A	N/A	N/A	N/A	N/A	2
Burns, 2013 [63]	51	TPB^g^	1; 3	18	N/A	1	N/A	2	N/A	N/A	1.5
Joseph, 2013 [51]	422	HBM	1; 2; 5; 6; 11	45	N/A	2	1	N/A	0.67	0	0.92
Lau, 2015 [59]	330	HBM; SCT; SET^h^; Transtheoretical change	1; 2; 5; 8; 11	45	N/A	0	0	N/A	0	0	0
Wiecha, 2015 [61]	58	SCT	1	9	1	N/A	0	N/A	0.25	0	0.31
Ahmed, 2016 [53]	98	Behavior change; SET; Motivational Theory	1; 2	18	N/A	0		1	N/A	0	0.33
Speck, 2016 [64]	44	SCT	1; 2; 3; 5; 6; 8; 11	64	N/A	2		2	N/A	N/A	2
Warren, 2016 [65]	12	SRT	1; 2; 3	27	N/A	0	2	0	N/A	N/A	0.67

^a^SRM: Self-Regulatory Model.

^b^SCT: Social Cognitive Theory.

^c^N/A: not applicable.

^d^SLT: Social Learning Theory.

^e^SRT: Self-Regulation Theory.

^f^HBM: Health Belief Model.

^g^TPB: Theory of Planned Behavior.

^h^SET: Self-Efficacy Theory.

#### Use of Theory

Data extracted included the theory(ies) reported in the intervention and the theoretical construct(s) addressed by the intervention. The Theory Coding Scheme (TCS) [52] was used to assess the extent to which theory had been applied. This instrument consists of 19 items, from which items 1 to 11 were relevant to this review, as items 12 to 19 do not measure the use of theory in the development of the interventions [48]. Items 1 to 11 assessed whether theory was mentioned in the paper, the use of theory to select participants, intervention techniques, or tailoring of the intervention and whether theoretical constructs or behavioral determinants were explicitly linked to intervention techniques [52]. For each study, a percentage score was calculated representing the proportion of relevant TCS items applied to the intervention ([number of TCS items applied divided by number of relevant TCS items] × 100).

#### Data Synthesis

Narrative synthesis was used to describe the impact of the interventions on the study outcomes and the application of theory in the development of the interventions. Pearson correlation coefficients were used to calculate the correlation between the effectiveness of interventions and the percentage score for the use of theory.

## Results

### Characteristics of the Interventions

From 1136 papers originally identified, 14 met the inclusion criteria (Figure 1). Multimedia Appendix 2 shows full details of the studies’ design and population characteristics. Of the 14 studies, 71% (10/14) reported on RCTs [51,53-61]), and 29% (4/14) were feasibility studies employing a pre-post design [62-65]. In all, 71% (10/14) of studies were undertaken in the United States. Studies included children (36%, 5/14) [54,56,61,62,65]), adolescents (14%, 2/14) [51,57]), adults (43%, 6/14); [53,55,59,60,63,64]), and mixed samples (7%, 1/14) [58]). Between 35% and 82% of the samples were female. Sample sizes ranged from 16 to 422 and included a total of 1856 participants. Multimedia Appendix 3 shows details of the type of digital platforms, the frequency of the interventions, details of the nondigital component, if applicable, and control conditions. None of the included studies incorporated measures to prevent dropout, with details of adoption and engagement with the interventions shown in Multimedia Appendix 2. A total of 2 studies involved patients in the development of the interventions [55,63].

### Effectiveness of Theory-Based Digital Interventions to Enhance Asthma Self-Management

#### Effect of Interventions on Behavioral Outcomes

##### Medication Adherence

Five studies (36%, 5/14) reported on adherence to preventer medications (Table 1), from which 3 studies measured adherence using electronic monitoring [55,61,62], and 2 used self-report [57,60]. This included 4 RCTs [55,57,60,61] and the single pre-post study [62]. A total of 3 studies reported a significant positive effect of the intervention on adherence [55,60,62]. Moreover, 2 studies were considered as having a partial effect, 1 reported controller medication adherence improved significantly from baseline for the subgroup of subjects with low (<75%) adherence on the intervention group only but also reported no significant differences in change between the intervention group and control group (*P*=.10) [61]; the other study [57] described their result as only marginally significant (*P*=.09; see Table 1).

##### Self-Management and Control

A total of 8 studies (57%; 8/14) measured self-management and control outcomes (Multimedia Appendix 4). Each of the 8 studies (5 RCTs and 3 pre-post studies) that measured self-management behavior and control [51,53-55,59,63-65] used a different measure. In terms of self-management, these included the Partners in Health Scale [63], a validated measure of self-management behaviors [54] and the Asthma Belief survey [65]. Asthma control was measured by the Asthma Control Questionnaire (ACQ) [59], the Asthma Control Test (ACT) [53,55,64], potential overuse of rescue fast-acting bronchodilators [53], indicators of uncontrolled asthma [51], and the Royal College of Physicians 3-questions screening tool [63]. In addition, 3 studies reported a significant positive effect of the intervention on self-management behavior [54], 2 studies reported a significant positive effect on asthma control [51,64], and 1 study [63] reported the intervention had a significant positive outcome on asthma control but not on self-management (Table 1). Only 1 [64] of the 2 pre-post studies showing a significant effect of the intervention on asthma control reported that the improvement of over 3 points on the ACT at 3 months was greater than the minimally important difference.

#### Effect of the Interventions on Clinical Outcomes

##### Clinical Markers of Health

A total of 8 studies (57%, 8/14: 7 RCTs and 1 pre-post study) reported on clinical markers of health (Multimedia Appendix 4). Measures included asthma symptoms, symptom days or symptom nights [51,54,57,58], forced expiratory volume [56], functional status measure [54], severe asthma exacerbation [58], worsening of asthma needing treatment changes [58], reported days of wheezing [61], peak expiratory flow rate [65], days of reliever use, and average daily dose of ICS [58]. Moreover, 3 studies reported a significant effect of the intervention on all of their clinical markers measured [54,57,65] (Table 1).

### Quality of Life

A total of 8 studies (57%, 8/14: 5 RCTs and 3 pre-post studies) reported on quality of life [53,55-58,63-65] (Multimedia Appendix 4). Validated measures included the Asthma Quality of Life Questionnaire (AQLQ) [55,64], the Paediatric Asthma Quality of Life Questionnaire [56,58,65], and the mini AQLQ [53]. Two studies developed a quality of life measure specific to their study [57,63]. In addition, 2 studies [63,64] reported a significant positive effect of the intervention on quality of life (Table 1). One study [53] reported a significant improvement from baseline to 3 months, but this effect was not significant at 6- and 9-month follow-ups.

#### Other Quality of Life Outcomes

A total of 5 studies (36%; 5/14) reported on factors influencing quality of life [51,57-59,61] (Multimedia Appendix 4). These included nights of sleep disturbance or patient awakening [58,61], days of activity limitation/restricted activity [51,57,58,61], number of school days missed [51,57,58,61], number of work days missed [59], days of changed plans [51,57], and number of days the patient had to slow down [61]. Two studies were partly effective in improving these outcomes [57,58] (Table 1). For example, although in 1 study [58], days of activity limitation and number of school days missed significantly decreased in the intervention group only (*P*<.01), there were no significant differences between the control and intervention groups.

#### Health Care Utilization

A total of 7 studies (50%, 7/14, all being RCTs) reported on health care utilization [51,53,54,57-59,61] (Multimedia Appendix 4). All measured the number of emergency department visits or hospitalizations over a given time. A total of 3 studies also reported the total number of urgent visits to a health care professional, general practitioner, or physician [58,59,61]. In all, 2 studies reported a significant decrease in hospitalizations following the intervention but no significant differences in emergency room visits [54,57]. One study found a significant decrease in emergency department annual visits in the intervention group but not for the number of hospitalizations or urgent visits to physicians [58]. A total of 4 studies did not find any significant effect of the intervention on health care utilization outcomes (Table 1).

### Theories That Have Been Applied to Intervention Development

Details of the theoretical basis of the intervention are shown in Table 1. Theories included Social Cognitive Theory [54,59,61,64], Health Belief Model [51,57,59], Theory of Planned Behavior [63]; Social Learning Theory [62], the Transtheoretical Model [57], the PRECEDE-PROCEED model [56], developmental and social support and learning theories [56], Behavior Change theory and Motivational theory [53], the Benefit-Risk Model of Health Behavior [55], and Self-Efficacy Theory [53,59]. A total of 5 of the interventions referenced the Self-Regulatory Model, Common Sense Model of Self-Regulation, Extended Common Sense Model of Self-Regulation, Illness Perceptions, or Necessity Concerns Framework in the development of the intervention [54,55,58,60,65].

### Theoretical Constructs That Have Been Addressed

Theoretical constructs/behavioral determinants specified within the models and addressed in the interventions included illness perceptions, which specifically explored identity, consequences, timeline, personal control, treatment control, concern, understanding, and emotional response to the illness [60]; beliefs about medicines were addressed in 3 interventions by targeting patients’ beliefs about the necessity of their medication and their concerns about taking their medication [53,55,60]. General control beliefs [54,64] and self-efficacy beliefs looked at how confident patients felt in areas such as self-management, which is taking medicines as prescribed, and self-awareness, which includes recognizing and acting on the symptoms [53,59,62,65].

### The Extent Theoretical Models Have Been Applied

**Figure 2:**
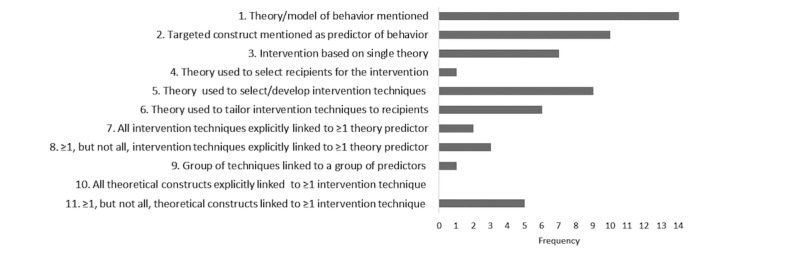
Frequency items from the Theory Coding Scheme used in the studies.

Responses to the TCS are shown in Table 1, and the frequency each item was reported in the studies is illustrated in Figure 2. In line with the study inclusion criteria, all studies (100%, 14/14) mentioned theory (item 1), and 10 studies (71%, 10/14) [51,53,55-57,59-62,64] mentioned a target construct as a predictor of behavior (item 2). Theory was explicitly used to select or develop intervention techniques (item 5) in 9 studies (64%, 9/14) [51,54-57,59,60,62,64]. A total of 7 studies (50%, 7/14) [54,55,60,62-65] referred to the application of a single theory rather than a combination of different theories (Item 3). A total of 6 studies (43%, 6/14) [51,54,57,60,62,64] used theory or predictors to tailor intervention techniques to participants (item 6). A total of 3 studies (21%, 3/14) [59,62,64] linked at least 1 intervention technique to a theory-relevant construct/ predictor (item 8), 2 studies (14%, 2/14) [60,62] linked all intervention techniques to at least 1 theory-relevant predictor (item 7), and 1 study [62] (7%, 1/14) linked a group of techniques to a group of clusters/predictors (item 9). Only 1 study (7%, 1/14) [60] screened or selected participants based on a particular score or level on a theory-relevant construct or predictor (item 4). No studies linked every theoretical construct within a stated theory to an intervention technique (item 10); however, 5 studies (36%, 5/14) [51,57,59,60,64] linked at least 1 theoretical construct to at least 1 intervention technique (item 11).

The use of theory as assessed by the TCS ranged from 9% to 73%. Three studies applied >60% of the different uses of theory based on the items of the TCS (6 items) [60,62,64] (Table 1). All 3 of these studies (100%) showed a significant positive effect of the intervention on all behavioral and clinical outcomes measured (average score 2.0; Table 1). Comparably, from the 11 studies that incorporated ≤60% of theory, no study was fully effective, but 3 interventions were partially effective (average score range 1.0-1.9) [54,57,63]. All other studies yielded average scores of <1.0. There was a significant correlation between the percentage of theory applied to the interventions and the effectiveness of the intervention (outcomes average score) (*r*=.657; *P*=.01). To assess whether sample size had an influence on the results, correlations were recalculated excluding Bartlett et al [62], with a small sample size of n=16. Results showed correlations were still highly significant, indicating theory and effectiveness were not biased by sample size (*r*=.581; *P*=.04). None of the studies reported using theory to promote engagement with the intervention.

**Figure 3:**
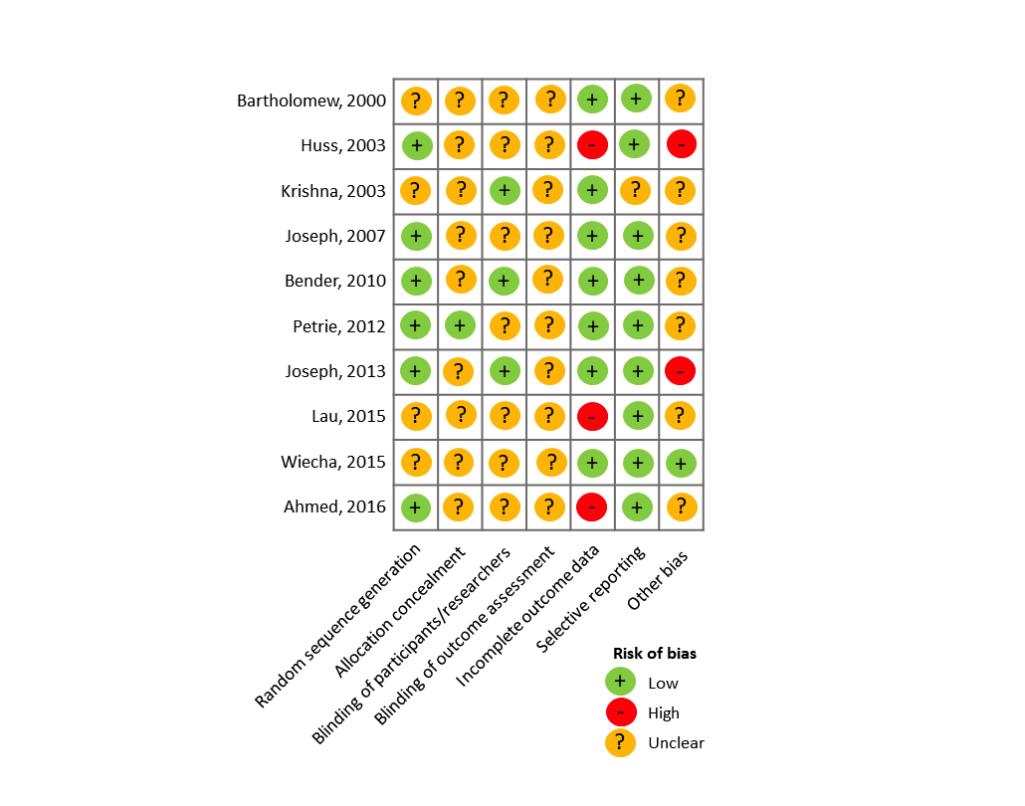
Risk of bias across interventions.

### Risk of Bias Assessment

Risk of bias assessment was performed on the 10 RCTs included in the review. The results are shown in Figure 3, and complete details are provided in Multimedia Appendix 5. A total of 6 studies reported using appropriate random sequence generation methods; of these all used computer-generated random allocation [51,53,55-57,60]. Four studies did not specify the method of randomization [54,58,59,61]. Only 1 study reported concealment of allocation [60], while this was unclear for the remaining 9 studies. None of the studies specified whether there had been blinding of the outcome assessment. Three studies were considered to have high risk of incomplete outcome data [53,56,59] due to high rates of attrition, whereas the remainder were considered to have low risk. A total of 9 studies were assessed as having low-risk of selective reporting, while this was unclear in 1 study [58] as measures had not been stated at the outset.

## Discussion

### Summary of Findings

This review identified 14 studies that evaluated theory-based digital interventions in RCTs or pre-post studies. A range of different theories had been used in the development of these interventions, most frequently Social Cognitive Theory, the Health Belief Model, and the Common-Sense Model of Self-Regulation [51,54,55,57-61,64,65]. The findings indicate that the use of psychological theory can enhance the effectiveness of digital interventions, as interventions that incorporated a more extensive use of theory were more likely to achieve successful outcomes. These findings are consistent with those of previous systematic reviews showing that digital self-management interventions can be effective at improving clinical outcomes in asthma [12,30,66] and suggest that theory-based interventions may be more effective than interventions that have not used theory in their development [46]. A previous meta-analytic review of internet- based interventions also found that extensive use of theory was associated with larger effect sizes on health behavior change [48].

To our knowledge, this is the first systematic review to examine the extent to which theory has been applied to the development of digital self-management interventions for asthma. We found substantial differences between studies in terms of their use of theory. Although most of the studies that mentioned theory referred to the use of theory in the development of their interventions, fewer studies explicitly reported the use of theory to select recipients for the intervention or indicated how they had linked intervention techniques, relevant constructs or predictors. Our findings suggest that interventions that incorporated these items in their development were more likely to be effective; however, only a small number of studies utilized these constructs. Further research is, therefore, required to ascertain how the application of theory in the development of interventions impacts their effectiveness.

Other factors, such as the delivery channel (eg, via different digital platforms), the context in which the intervention is delivered (eg, via hospital or routine assessments), and the type of user (eg, children vs adults) may also influence outcomes. The fact that interventions that applied theory to a similar extent could have varying degrees of effectiveness implies that the use of theory is necessary, but not sufficient, for a successful intervention.

### Strengths and Limitations

The strengths of this review include the systematic approach, inclusion of a range of interventions focusing on many different self-management behaviors, and the use of a reliable instrument to determine the extent to which theory had been used to inform the design of the interventions. The heterogeneity in outcomes measured precluded the use of meta-analysis, therefore, we were not able to determine the size of the effect. Although the findings indicate an increasing number of researchers are utilizing theory in the development of digital interventions for asthma, there were insufficient numbers of studies referencing each theoretical model to determine whether any one theory showed promise over another.

Limitations of the individual studies included a lack of information describing the interventions. Often it was not possible to determine which behavioral determinants had been targeted or how they had been addressed by the intervention. This could be improved in future studies through the use of a framework such as the Template for Intervention Description and Replication checklist [67] to describe the intervention. This would not only aid replication but also allow a more reliable and thorough assessment of the process by which digital self-management interventions exert their effect. In addition, there was a lack of information on methods of randomization and concealment in many of the studies, meaning that the risk of bias was often unclear. Eysenbach [58] stated there is a need to address the “law of attrition,” which relates to the dropout and nonengagement in electronic health users. A high dropout rate was observed in the interventions included within this review [53,59,60]. However, none of the included studies incorporated measures to engage participants in the intervention and prevent dropout, and none of the studies mentioned they used theory to increase engagement with the interventions. The short duration of some studies means that individual studies may have been underpowered or overpowered for individual outcomes. Further research is needed to explore how theory could specifically target engagement behavior to achieve effective engagement.

### Implications of Our Findings for Clinical Care and Future Research

Our findings suggest that theory-based digital interventions to enhance asthma self-management can be effective at improving adherence and self-management and that more extensive use of theory in the development and application of digital interventions for asthma self-management may enhance their effectiveness. However, although a number of theories have been applied to the development of asthma digital interventions, it is not clear whether any particular theory is more effective. Furthermore, most studies lack details on the theoretical constructs used and behavioral determinants addressed by the intervention, and whether or how these have been applied to the design or application of the intervention. The systematic recording and reporting on the use of theory in the development of future interventions is, therefore, important. It is not sufficient to merely state theory has been used; there should be specific reference to exactly how it has informed the design of the intervention. The TCS can be used to inform the design of an intervention, ensuring that the theoretical basis of an intervention is adequately and clearly described so that the use of theory can be evaluated.

## Appendix

Multimedia Appendix 1 Search terms. Terms within columns were combined using the Boolean "OR" operator, terms between columns were then combined with "AND," that is, papers were retrieved if the title/abstract/keywords contained at least one term from each column.

Multimedia Appendix 2 Study characteristics: Study design, population characteristics, and intervention engagement.

Multimedia Appendix 3 Details of mode of delivery of the intervention.

Multimedia Appendix 4 Behavioral (adherence, self-management, and control) and clinical outcomes of studies. Randomized control trials’ (RCT) values reported in this table refer to the differences between intervention (IG) and control groups (CG); values for pre-post intervention studies report changes from baseline.

Multimedia Appendix 5 Risk of Bias table.

## Abbreviations

ACT: Asthma Control Test
AQLQ: Asthma Quality of Life Questionnaire
HBM: Health Belief Model
ICS: inhaled corticosteroids
mHealth: mobile health
RCT: randomized controlled trials
SCT: Social Cognitive Theory
SET: Self-Efficacy Theory.
SLT: Social Learning Theory
SMS: short message service
SRM: Self-Regulatory Model
SRT: Self-Regulation Theory
TCS: Theory Coding Scheme
TPB: Theory of Planned Behavior
