# Supplementary material for: Theory-Based Digital Interventions to Improve Asthma Self-Management Outcomes: Systematic Review
Source: J Med Internet Res. 2018 Dec 12;20(12):e293. doi: 10.2196/jmir.9666 (PMC6306620; doi:10.2196/jmir.9666)
Supplement: Multimedia Appendix 1 [file jmir_v20i12e293_app1.pdf]

Multimedia Appendix 1 - Search terms. Terms within columns were combined using the Boolean ‘OR’ operator, terms between columns were then combined with ‘AND’ – i.e. papers were retrieved if the title/abstract/ keywords contained at least one term from each column.

digital	adherence	intervention	asthma
online	persistence	program*	
Internet	engagement	outcome	
wearable*	self-management		
mobile	perception*		
smart	psycholog*		
app	belief*		
application			
Monitor*			
electronic			
automated			
feedback			
SMS			
text			
IVR			
voice recognition			
text			
eHealth			

** denotes truncation*
