# Supplementary material for: Theory-Based Digital Interventions to Improve Asthma Self-Management Outcomes: Systematic Review
Source: J Med Internet Res. 2018 Dec 12;20(12):e293. doi: 10.2196/jmir.9666 (PMC6306620; doi:10.2196/jmir.9666)
Supplement: Multimedia Appendix 2 [file jmir_v20i12e293_app2.pdf]

Multimedia Appendix 2 - Study characteristics: Study design, population characteristics and intervention engagement.

CG: control group; FU: follow-up; IG: intervention group; NS: not stated; RCT: randomised control trial.

First author, year	Country	Study design	Sample Size	Female (%)	Mean age (SD) or range	Duration of intervention	Intervention engagement
Bartholomew, 2000 [54]	U.S.	RCT	IG (n=70)<br>CG (n=63)	35	IG 11.62 (2.15)<br>CG 11.30 (2.56)	4 -15.6 months	60% followed programme directions
Bartlett, 2002 [62]	U.S.	Pre/post	S1: IG (n=5)<br>S2: IG (n=11)	NS	7-12	Study 1: 4 weeks<br>Study 2: 5 weeks	NS
Huss, 2003 [56]	U.S.	RCT	IG (n=56)<br>CG (n=45)	44	9.6 (1.8)	12 weeks	47 children lost to FU
Krishna, 2003 [58]	U.S.	RCT	IG (n=107)<br>CG (n=121)	35	0-17	12 months	Caregivers of 0-6-year-olds: 40-100% completed visit 3; 50% of users completed ≥40% of vignettes. 7-17-year-old group: 50% completed ≥58% of the program
Joseph, 2007 [57]	U.S.	RCT	IG (n=162)<br>CG (n=152)	63.4	15.3 (1.0)	180 days;<br>(FU: 12-months)	NS
Bender, 2010 [55]	U.S.	RCT	IG (n=25)<br>CG (n=25)	IG 60<br>CG 68	IG 39.6 (12.8)<br>CG 43.5 (14.3)	10 weeks	NS
Petrie, 2012 [60]	New Zealand	RCT	IG (n=73)<br>CG (n=74)	68	16-45	18 weeks;<br>(FU: 9-months)	Consent form returned: 68%; Last FU completed: 75% (n=124)
Burns, 2013 [63]	Australia	Pre-post	IG (n=51)	66.7	62.5 (6.73)	3 months	NS
Joseph, 2013 [51]	U.S.	RCT	IG (n=204)<br>CG (n=218)	NS	15.6	12 months	88.4% completed all sessions; 90% completed 12-month FU
Lau, 2015 [59]	Australia	RCT	IG (n=154)<br>CG (n=176)<br>Case analysis:<br>IG (n=56)<br>CG (n=97)	IG 85<br>CG 79.5<br>Case analysis:<br>IG 79<br>CG 84	IG 40 (SD 14)<br>CG 39 (SD 13)<br>Case analysis:<br>IG 46 (14)<br>CG 41 (14)	12 months	80.5% accessed the intervention 0-1 times; only 1 accessed the intervention ≥10 times; participants lost to FU (IG 98; CG 79)
Wiecha, 2015 [61]	U.S.	RCT	IG (n=37)<br>CG (n=21)	IG 40.5<br>CG 42.9	IG 11.9 (2.0)<br>CG 12.9 (3.0)	6 months	At 6-months, CG retained 14 (66.7%) of enrolled subjects; IG 28 (75.7%); the other subjects were lost to FU before the 6-month end-point
Ahmed, 2016 [53]	Canada	RCT	IG (n=47)<br>CG (n=51)	IG 68<br>CG 65	18-70	6 months;<br>(FU: 3-months)	4 never logged-in. Logins more frequent up to 4 weeks (mean 12; SD 8); declined after (mean 36; SD 42)
Speck, 2016 [64]	U.S.	Pre-post	IG (n=44)	77	24.7 (3.6)	6 weeks;<br>(FU: 3-months)	79.5% utilised the diary at least once
Warren, 2016 [65]	U.S.	Pre-post	IG (n=12)	NS	12-14	13 weeks	3 of the original 14 dropped out within the first 2 weeks
