# Supplementary material for: Theory-Based Digital Interventions to Improve Asthma Self-Management Outcomes: Systematic Review
Source: J Med Internet Res. 2018 Dec 12;20(12):e293. doi: 10.2196/jmir.9666 (PMC6306620; doi:10.2196/jmir.9666)
Supplement: Multimedia Appendix 3 [file jmir_v20i12e293_app3.pdf]

Multimedia Appendix 3 - Mode of delivery of the intervention. EM: Electronic monitor; FD: Fully digital; FTF: face-to-face; i: individual; IVR: interactive voice response; NS: not stated; PD: partly digital; SMS: short message service.

Study	FD/PD	Digital platform	Frequency of intervention	Non-digital component	Control condition
Bartholomew, 2000 [54]	FD	CD-ROM computer game	During routine clinician's visits	NS	Usual care
Bartlett, 2002 [62]	PD	EM	Weekly	FTF (i)	NS
Huss, 2003 [56]	PD	Interactive Game	NS	FTF and telephone (i)	Usual care and computer game
Krishna, 2003 [58]	PD	CD-ROM	During routine clinician's visits	FTF & paper-based (i)	Usual care
Joseph, 2007 [57]	FD	Web	4 sessions	NS	General asthma websites
Bender, 2010 [55]	FD	EM and IVR	Monthly	NS	NS
Petrie, 2012 [60]	FD	SMS (one-way)	Weeks:<br>1-6=2 SMS/day;<br>7-12=1 SMS/day;<br>13-18=3 SMS/week	NS	Usual care
Burns, 2013 [63]	FD	Web	N/A	NS	NS
Joseph, 2013 [51]	FD	Web	4 sessions	NS	General asthma websites
Lau, 2015 [59]	FD	Web (and email reminders)	NS	NS	Web page
Wiecha, 2015 [61]	FD	Web	NS	NS	Asthma education manual; peak flow meter; usual care
Ahmed, 2016 [53]	PD	Web	Logged into portal weekly	Telephone	Usual care
Speck, 2016 [64]	FD	Web (and email reminders)	Every 3 days participants asked to log in and complete assignment	Telephone	NS
Warren, 2016 [65]	PD	Internet-enabled mobile device	3 days/week	Group work	NS
