# Supplementary material for: Theory-Based Digital Interventions to Improve Asthma Self-Management Outcomes: Systematic Review
Source: J Med Internet Res. 2018 Dec 12;20(12):e293. doi: 10.2196/jmir.9666 (PMC6306620; doi:10.2196/jmir.9666)
Supplement: Multimedia Appendix 4 [file jmir_v20i12e293_app4.pdf]

Multimedia Appendix 4. Behavioural (adherence, self-management and control) and clinical outcomes of studies. Randomised control trials' (RCT) values reported on this table refer to differences between intervention (IG) and control groups (CG), values for Pre-post intervention studies report changes from baseline.

Author, Year	Adherence	Self-management and control	Clinical markers of health	QoL[b]	Other QoL outcomes	Healthcare utilisation
Bartholomew, 2000 [54]	N/A[a]	*Child self-management** - IG with higher pre-test scores did CG with higher pre-test scores (t(127)=2.1; *P*=0.03)	*Symptoms** (parent questionnaire) - total symptom score intervention effect (t(116)=-1.96; *P*=0.05)<br>*The functional status measure** (reported by caretaker) - pre and post-test F(1,107)5.30; *P*=0.02) but difference depended upon time between pre and post-test (F(1,107)7.05; *P*=0.01), the shorter the time, the smaller the advantage for the IG			*Hospitalisation**- (F(1, 111) 6.28; *P* =0.01)<br>*ED[c] visits* – NSD[d] *P*>0.05
Bartlett, 2002 [62]	*Adherence** (electronic metered monitors) - Study 2: By week 5, the number of children using their medications appropriately doubled from 29% to 54% (*P* =0.004), underutilisation decreased from 51% to 25% (*P* =0.02). Number of children reporting days with no medication use dropped from 28% at baseline to 15% by week 5 (*P* =0.009)					
Huss, 2003 [56]			*Percentage of predicted Forced Expiratory Volume* (FEV) - NSD (*P*=0.93)	*Paediatric Asthma Quality of Life* – NSD (*P*=0.48)		
Krishna, 2003 [58]			*Asthma symptoms**: IG significantly greater decrease in days with asthma symptoms, (χ^2 = 6.65, *P*<0.01)<br>*Average daily dose of ICS** - At visit 3, significantly lower in IG (434 µg) compared with CG (754 µg), (*P* <0.01)<br>*Days of quick relief medicine use* – NSD (*P* >0.05)	*QOL scores* - NSD (χ^2= 2.51; *P*< 0.11)	*Nights of sleep disturbance* - *Both CG and IG showed significant reductions from visit 1 to visit 3 (*P* <0.05); NSD between groups<br>*Days of activity limitation* - *Decreased significantly between visits in the IG alone (*P*<0.01); NSD between groups	*Urgent visits to physicians* – NSD between groups<br>*ED annual visits** - IG significantly greater decrease (1.93 vs 0.62 per year) (χ^2 = 5.07, *P* <0 .01)<br>*Hospitalisations* – NSD between groups

Author, Year	Adherence	Self-management and control	Clinical markers of health	QoL[b]	Other QoL outcomes	Healthcare utilisation
					Number of school days missed - *Decreased significantly between visits in the IG alone (*P*<0.01); NSD between groups	
Joseph, 2007 [57]	*Adherence* - Significantly fewer IG students with controller medication were adherent compared with CG: Positive changes in controller medication adherence were of borderline significance (*P*=0.09)		*Number of symptom days in the last 2 weeks* * - IG reported significantly fewer symptom days than CG (*P*=0.003)<br>*Symptom nights** - IG reported significantly fewer symptom nights (*P* = 0.009)	*QOL scores* - NSD; all *P*>0.05	*Days of restricted activity**- IG reported significantly fewer days (*P* = 0.01)<br>*School days missed in last 30 days**- IG reported significantly fewer days (*P*=0.006)<br>*Days of changed plans* - lower for IG compared with CG but NSD (*P*=0.17)	*ED visits for asthma* - lower for IG compared with CG but the difference was only marginally significant (p=0.08)<br>*Hospitalisations in the last 3 months** - significantly lower for IG when compared with CG (*P* = 0.01)
Bender, 2010 [55]	*Adherence** – Mean ICS adherence was higher in IG than in the CG by a margin of 64.5% to 49.1% (F = 9.66; *P*=0 .0032)	*Asthma control test (ACT)* – NSD for ACT scores (*P*=0.53)		*Asthma QoL questionnaire (AQOL) scores* -NSD for any of the AQLQ scores, or total (*P*=0.419)		
Petrie, 2012 [60]	*Adherence** - Baseline mean adherence: 54% (SD = 31.8%) in the CG and 56.5% (SD = 35.3%) in IG, t(213) = -0.53, *P* = 0.60. No overall time effect, but a significant group effect (F(1,122) = 9.35, *P* = .003), and a significant group by time effect (F(5) = 2.27, *P* <0.05). Average self-reported adherence over all time points in CG was 43.2% (SD = 26) and the IG was 57.8% (SD = 27.1), t(122) = -3.06, *P* = 0.003). Proportions with average adherence of ≥80% for the CG was 10.6% and for IG 25.9%. The difference between the two groups was 15.3%, *P*=0.034					
Burns, 2013 [63]		*Asthma control (RCP3 tool)** - significantly improved (pre-mean 2.16; post 1.34) (t = 4.95, *P*<0.01)		*QoL (own scale)** - Significantly improved		

Author, Year	Adherence	Self-management and control	Clinical markers of health	QoL[b]	Other QoL outcomes	Healthcare utilisation
		Asthma self-management efficacy - decreased (pre-mean 19.36; post 28.20) (t=4.06, *P*<0.01)		(pre-mean 3.20; post 2.54) (t =2.61, *P*=0.012)		
Joseph, 2013 [51]		*Indicators of uncontrolled asthma** - comparisons were significant for the outcome of >8 symptom-days in the past 30 days (or >2 symptom-days/week in the past 30 days) (*P* =0.006), and >4 days of restricted activity in the past 30 days (*P* = 0.010)	*At 12 months (all):*<br>*Symptom days** - (*P*=0.019)<br>*Symptom nights* – NSD (*P*=0.82)<br>*At 12 months (for moderate to severe asthma):*<br>*Symptom days** - (*P*=0.013)<br>*Symptom nights* – NSD (*P*=0.210)		At 12 months (all):<br>*School days missed because of asthma* – NSD (*P* = 0.25)<br>*Days of restricted activity* – NSD (*P* = 0.14)<br>*Days had to change plans* – NSD (*P* = 0.96)<br>At 12 months (for moderate to severe asthma):<br>*School days missed because of asthma** - (*P* =0 .007)<br>*Days of restricted activity**- (*P* =0 .025)<br>*Days had to change plans* - (*P* =0.064)	*Medical care use (for all):*<br>*ED visits* – NSD (*P* = 0.92)<br>*Hospitalisations* -NSD (*P* =0 .66)<br>*Medical care use (for moderate to severe asthma):*<br>*ED visits* - NSD - (*P* =0 .95)<br>*Hospitalisations*-NSD (*P* =0.47)
Lau, 2015 [59]		*Asthma control Questionnaire (ACQ)* – NSD (*P*=0.25)	*Severe asthma exacerbation* – NSD (*P*=0.37)<br>*Worsening of asthma that required treatment changes* – NSD (*P*=0.29)		*Missing one or more days from work or study due to asthma* – NSD (*P*=0.26)	*Emergency visit to healthcare professional* - NSD (*P*=0.96)<br>*Emergency visit to ED* - NSD (*P*=0.88)<br>*Emergency visit to GP or respiratory physician* – NSD (*P*=0.96)
Wiecha, 2015 [61]	*Adherence* -<br>*Subgroup of subjects with low (<75%) controller medication adherence at baseline: controller medication adherence at 6 months improved significantly only in IG (IG mean baseline 16.3; mean change at 6 months +29.8; *P* =0.01);<br>No IG CG difference (*P* = 0.10)		*Reported days of wheezing* **–** NSD (*P*=0.10)		*Days had to slow down* - NSD (*P*=0.79)<br>*Days limited activity* - NSD (*P*=0.14)<br>*Patient awakening** - improved only in the IG: −0.8 days change per 2 weeks at 6 months; but NSD in change (*P*=0.66)<br>*Days missed from school* - NSD (*P*=0.31)	*ED or acute visits to a physician* -NSD (*P*= 0.64)
Ahmed, 2016 [53]		*Asthma control (evaluated by potential overuse (yes/no) of rescue fast-acting bronchodilator)* - NSD between groups over time for poor control status. There was no significant effect of group, time, or group by time		*Mini asthma QoL Questionnaire (MAQLQ)** - significant improvement in MAQLQ score between baseline and 3 months for the IG (mean change 0.67, 95% CI 0.36 to 0.98).		*Asthma repeated visits to ED or hospitalisations* - NSD (P=0.42)

Author, Year	Adherence	Self-management and control	Clinical markers of health	QoL[b]	Other QoL outcomes	Healthcare utilisation
				NSD for the intervention group between 3-6 months (mean change –0.01, 95% CI –0.35 to 0.32) or 6-9 months (mean change –0.12, 95% CI –0.46 to 0.22)		
Speck, 2016 [64]		*Asthma Control Test (ACT)** - improved from 16.1 to 19.3 (*P* < 0.01). Average ACT score 3 months after the program was 19.4 v 3.6 and improved significantly from baseline (*P* <0.001)		*Mini Asthma Quality of Life Questionnaire (AQLQ)** - improved from 4.0 to 5.1 (*P* < 0.01). 3 months after program completion average score of 5.2 ±1.3 (*P*<0.001), which was also significantly increased from baseline		
Warren, 2016 [65]		*Self-management (self-care practices)* - (z=1.18; *P*=0.21)	*Lung function (Peak expiratory flow rate)**- (z=3.41; *P*<0.01)	*Paediatric Asthma Quality of Life Questionnaire (PAQLQ)* -(z=1.37; *P*=0.17)		

[a]N/A: not applicable; [b]QoL: Quality of Life; [c]ED: emergency department; [d]NSD: not significant difference (*P*>0.05); * Significant outcome (p<0.05)
