# Supplementary material for: Theory-Based Digital Interventions to Improve Asthma Self-Management Outcomes: Systematic Review
Source: J Med Internet Res. 2018 Dec 12;20(12):e293. doi: 10.2196/jmir.9666 (PMC6306620; doi:10.2196/jmir.9666)
Supplement: Multimedia Appendix 5 [file jmir_v20i12e293_app5.pdf]

Multimedia Appendix 5 - Risk of Bias Table. CG: control group; IG: intervention group; NS: not stated.

Trial	Random sequence generation	Allocation concealment	Blinding of participants/ researchers	Blinding of outcome assessment	Incomplete outcome data	Selective reporting	Other bias
Bartholomew, 2000 [54]	*Unclear*	*Unclear*	*Unclear*	*Unclear*	*Low*<br>38 lost to follow-up; attrition did not differ between IG & CG	*Low*<br>All pre-specified outcomes reported	NS
Huss, 2003 [56]	*Low*<br>Allocation to CG and IG by computer generating random numbers	*Unclear*	*Unclear*	*Unclear*	*High*<br>Lost to follow-up: 47	*Low*<br>All pre-specified outcomes reported	*High*<br>Small sample size
Krishna, 2003 [58]	*Unclear*	*Unclear*	*Low*<br>Pulmonologists caring for the participants were blind to participant enrolment and group assignment	*Unclear*	*Low*<br>3 families declined to participate. 17 subjects were excluded at the request of the participants themselves or lack of data. One IG child was excluded from analysis because of suspected diagnosis of Munchausen by proxy; final sample 228 children	*Unclear*<br>Outcomes were not pre-specified	NS
Joseph, 2007 [57]	*Low*<br>A random number generator was used within each unique stratum	*Unclear*	*Unclear*	*Unclear*	*Low*<br>No difference between groups completing follow up	*Low*<br>All pre-specified outcomes were reported	NS
Bender, 2010 [55]	*Low*<br>Group assignment determined by randomisation table generated before study initiation	*Unclear*	*Low*<br>Investigators remained blind to treatment until the final data set was completed	*Unclear*	*Low*<br>Outcome data were complete for all study participants	*Low*<br>All pre-specified outcomes were reported	NS
Petrie, 2012 [60]	*Low*<br>Randomisation sequence generated by computer program	*Low*<br>Allocation concealed in consecutively numbered sealed envelopes	*Unclear*	*Unclear*	*Low*<br>68% screened returned the consent form. Drop-out rates were not significantly different between groups	*Low*<br>All pre-specified outcomes were reported	NS

Trial	Random sequence generation	Allocation concealment	Blinding of participants/ researchers	Blinding of outcome assessment	Incomplete outcome data	Selective reporting	Other bias
Joseph, 2013 [51]	*Low*<br>A random number generator was used within each unique stratum	*Unclear*	*Low*<br>Research staff, statisticians, and investigators were blinded to group assignment	*Unclear*	*Low*<br>90% completed follow up survey (IG). Exclusion of 2 outliers	*Low*<br>All pre-specified outcomes were reported	*High*<br>Baseline variables suggested slightly higher baseline morbidity for treatment students. Study design did not include randomisation within the treatment group for receipt of submodules
Lau, 2015 [59]	*Unclear*	*Unclear*	*Unclear*	*Unclear*	*High*<br>This study suffered from moderate to high rates of attrition in the IG (64%) and CG (45%)	*Low*<br>All pre-specified outcomes were reported	NS
Wiecha, 2015 [61]	*Unclear*	*Unclear*	*Unclear*	*Unclear*	*Low*<br>At the 6-month end-point, the CG retained 14 (66.7%) of enrolled subjects, and IG retained 28 (75.7%) of subjects. No significant dependence of drop out on the outcome values	*Low*<br>Continuous variables of interest were not always normally distributed; performed both non-parametric and parametric analyses. Only parametric analyses were reported	*Low*<br>Modest sample size resulting in limited statistical power
Ahmed, 2016 [53]	*Low*<br>Treatment allocation was done by random permutation within blocks with block sizes of 4 and 6 using a computerised algorithm	*Unclear*	*Unclear*	*Unclear*	*High*<br>Attrition of 37%, dropout attrition rate was >5 times higher in the IG compared with the CG	*Low*<br>All pre-specified outcomes were reported	NS
